# Frequency and molecular characterisation of *Entamoeba histolytica*, *Entamoeba dispar*, *Entamoeba moshkovskii*, and *Entamoeba hartmanni* in the context of water scarcity in northeastern Brazil

**DOI:** 10.1590/0074-02760150383

**Published:** 2016-02

**Authors:** Deiviane Aparecida Calegar, Beatriz Coronato Nunes, Kerla Joeline Lima Monteiro, Jéssica Pereira dos Santos, Helena Keiko Toma, Tais Ferreira Gomes, Marli Maria Lima, Márcio Neves Bóia, Filipe Anibal Carvalho-Costa

**Affiliations:** 1 Fundação Oswaldo Cruz, Instituto Oswaldo Cruz, Laboratório de Biologia e Parasitologia de Mamíferos Silvestres Reservatórios, Rio de Janeiro, RJ, Brasil; 2 Fundação Oswaldo Cruz, Instituto Oswaldo Cruz, Laboratório de Epidemiologia e Sistemática Molecular, Rio de Janeiro, RJ, Brasil; 3 Fundação Oswaldo Cruz, Escritório Técnico Regional Fiocruz Piauí, Brasil; 4 Universidade Federal do Rio de Janeiro, Laboratório de Diagnóstico Molecular e Hematologia, Rio de Janeiro, RJ, Brasil; 5 Fundação Oswaldo Cruz, Instituto Oswaldo Cruz, Laboratório de Ecoepidemiologia da Doença de Chagas, Rio de Janeiro, RJ, Brasil

**Keywords:** parasites, protozoa, entamoebiasis, epidemiology, Brazil

## Abstract

This study aimed to estimate the frequency, associated factors, and molecular characterisation of *Entamoeba histolytica*, *Entamoeba dispar*, *Entamoeba moshkovskii*, and*Entamoeba hartmanni* infections. We performed a survey (n = 213 subjects) to obtain parasitological, sanitation, and sociodemographic data. Faecal samples were processed through flotation and centrifugation methods.*E. histolytica*, *E. dispar*, *E. moshkovskii*, and *E. hartmanni* were identified by nested-polymerase chain reaction (PCR). The overall prevalence of infection was 22/213 (10.3%). The infection rate among subjects who drink rainwater collected from roofs in tanks was higher than the rate in subjects who drink desalinated water pumped from wells; similarly, the infection rate among subjects who practice open defecation was significantly higher than that of subjects with latrines. Out of the 22 samples positive for morphologically indistinguishable*Entamoeba* species, the differentiation by PCR was successful for 21. The species distribution was as follows: 57.1% to *E. dispar*, 23.8% to *E. histolytica*, 14.3% to*E. histolytica* and *E. dispar*, and 4.8% *E. dispar* and *E. hartmanni*. These data suggest a high prevalence of asymptomatic infection by the group of morphologically indistinguishable *Entamoeba histolytica*/*dispar*/*moshkovskii*complex and *E. hartmanni* species. In this context of water scarcity, the sanitary and socioenvironmental characteristics of the region appear to favour transmission.

Intestinal protozoan infections are closely related to a lack of proper sanitation and environmental contamination with faecal matter. Thus, their prevalence is higher in specific environmental scenarios that occur most often in developing countries (Ojha et al. 2014, Turkeltaub et al. 2015). Amoebiasis is a potentially severe and life threatening infection caused by enteric protozoa (Ralston & Petri Jr 2011, Skappak et al. 2014), most commonly *Entamoeba histolytica*, which is distributed worldwide (WHO 1997, Jackson 1998). The motile (trophozoite) form of*E. histolytica* inhabits the human colon where it multiplies and differentiates into cysts that are released into the environment. In turn, these cysts are responsible for transmitting the infection to another host *via* the faecal-oral route. The parasite invades the intestinal mucosa and causes many forms of invasive disease, including dysentery (Lin & Kao 2013). The parasite also exhibits bloodborne spreading and causes extraintestinal lesions, mainly liver abscesses (Wuerz et al. 2012). The latter form occurs only rarely. Invasive disease occurs when virulent trophozoites disrupt the mucoepithelial barrier by crossing the mucus layer, thereby damaging intestinal cells. This damage leads to inflammation and, consequently, dysentery (Thibeaux et al. 2013). Nevertheless, the majority of infections seem to be asymptomatic (Chacín-Bonilla 2013).

The existence of nonpathogenic indistinguishable *E. histolytica*/*Entamoeba dispar*/*Entamoeba moshkovskii*complex and *Entamoeba hartmanni* organisms capable of inhabiting the human intestine as commensals has been recognised for many decades. For instance, in 1926, Brumpt proposed the existence of *E. dispar*, a species indistinguishable by light microscopy from *E. histolytica*. However, *E. dispar* exhibits distinct physiological, biochemical, and ultrastructural characteristics, the latter of which have been described more recently (Goldman 1969, Jackson 1998, Pimenta et al. 2002). Another four-nucleated morphologically identical organism, *E. moshkovskii*, has been observed in sewage as a free-living amoeba, but is also capable of colonising the human intestine (Tshalaia 1941, Ngui et al. 2012). In addition, differential diagnosis should also consider the nonpathogenic species *E. hartmanni*, which can be distinguished from *E. histolytica*by its small cyst size (5-10 µm in diameter). In contrast, the diameter of *E. histolytica* cysts ranges from 12-14 µm (Brumpt 1949).

More recently, dysentery and extraintestinal disease have been proposed to be potentially associated with *E. dispar* and *E. moshkovskii* (Parija & Khairnar 2005, Costa et al. 2010). These findings complicated our understanding of the pathogenic behaviour and public health importance of indistinguishable *E. histolytica/E. dispar/E. moshkovskii* complex and *E. hartmanni* parasites (Oliveira et al. 2015).

Vast rural areas in northeastern Brazil are characterised by deficits in sanitation infrastructure. Moreover, improper disposal of waste occurs frequently. These semiarid regions are also subjected to water stress due to prolonged droughts. Therefore, alternative water management approaches have been applied in this region (Rasella 2013). In this context, specific epidemiological scenarios associated with water scarcity could favour transmission of enteric pathogens. For example, water must be stored for many months during the dry season.

This study aimed to use molecular techniques to estimate the frequencies of infection with *E. histolytica*, *E. dispar*, *E. moshkovskii*, and *E. hartmanni* in a population subjected to water scarcity in the Northeast Region of Brazil*.* This study also aimed to identify factors associated with these infections.

## SUBJECTS, MATERIALS AND METHODS

**Fig. 1:**
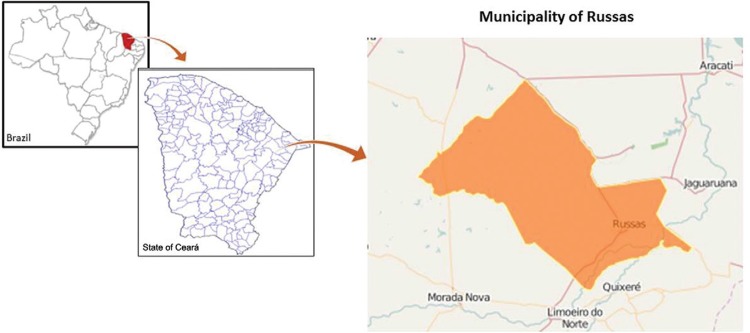
: map of the study area (Russas, state of Ceará, Brazil, 2013).

*Study area and population* - This study was performed in Russas, a municipality located 165 km from Fortaleza, the capital of the state of Ceará (Fig. 1). This region belongs to the semiarid region of northeastern Brazil, in the *Caatinga* biome. Russas has 74,243 inhabitants and a total area of 1,588 km^2^. The study included four rural communities in the municipality: Riacho do Barro (132 inhabitants), Timbaúba do Pitingão (109 inhabitants), Barracão (315 inhabitants), and Patos de Tito (54 inhabitants). Russas has a hot, dry climate and is subjected to prolonged droughts. The rainy season typically extends from December-June (annual rainfall in 2013 = 418 mm, mean annual rainfall = 792.6 mm). Nevertheless, seasonal rains have been reduced in the last few years and the region has been subjected to severe drought during the field work periods.

*Study design and sampling strategy* - We performed a cross-sectional survey from August-September 2013. The survey included 213 subjects (70 families): 53 subjects (18 families) from Timbaúba do Pitingão, 28 subjects (9 families) from Riacho do Barro, 119 subjects (38 families) from Barracão and 13 subjects (5 families) from Patos do Tito. Therefore, our study included 35% of the 610 residents in the four communities. We designed our sampling strategy specifically to include all households with children. During domicile visits, researchers distributed bottles without preservatives for faeces collection and obtained sanitation and sociodemographic data. In addition, the field team investigated whether the residents presented symptoms consistent with amoebiasis, such as diarrhoea, presence of mucus, pus, and/or blood in the stool, and abdominal pain, among others. The baseline characteristics of the study subjects are presented in Table I. Stool samples were collected the next day at each household and were transported to the field laboratory under refrigeration (4ºC). The rates of *E. histolytica*, *E. dispar*, *E. moshkovskii,* and *E. hartmanni*detection in distinct sociodemographic settings were compared using Fisher’s exact test. Statistical significance was established at p < 0.05.

**TABLE I:** Sociodemographic characteristics of the studied population, Russas, state of Ceará, Brazil, 2013

Characteristics	n (%)
Gender	
Male	106 (49.8)
Female	107 (50.2)
Age group (years)	
0-4	18 (8.4)
5-9	27 (12.7)
10-14	37 (17.4)
15-19	14 (6.6)
> 19	117 (54.9)
Community	
Barracão	119 (55.9)
Patos do Tito	13 (6.1)
Riacho do Barro	28 (13.1)
Timbaúba do Pitingão	53 (24.9)
Income strata	
Extreme poverty (< US$^*a*^ 17)	20 (9.4)
Poverty (US$ 17-34)	27 (12.7)
Not poor (> US$ 34)	166 (77.9)
Source of drinking water	
Desalinated brackish water from wells	138 (64.8)
Rain water stored in cisterns	56 (26.3)
Other	19 (8.9)
Sanitation facilities	
Latrine	166 (77.9)
Open defecation	47 (22.1)

*a*: US$ 1.00 = R$ 4,00 (22 September 2015).

*Laboratory procedures* - Initially, faecal samples were processed through the zinc sulphate flotation (Faust technique) and the formalin-ethyl-acetate centrifugation (modified Ritchie technique) methods (Faust et al. 1938, Young et al. 1979). For the Faust technique, 7 mL of gauze-filtered faecal suspension was spun by centrifugation and the resultant pellet was re-suspended in zinc sulphate solution (1,180 g/mL). The suspension was shaken and spun by centrifugation again, after which the resultant supernatant was examined by light microscopy. For the Ritchie method, gauze-filtered faecal suspensions were spun by centrifugation and the resultant pellets were re-suspended in 5 mL of water and 3 mL of ethyl-acetate was added to each suspension. The sedimented matter was examined by light microscopy. It was not possible to perform permanent smear staining for light microscopy or to measure amoebae cysts in the field laboratory; thus, *E. hartmanni* could not be distinguished from *E. histolytica*, *E. dispar*, and *E. moshkovskii*. Faecal samples were cryopreserved and transported to the city of Rio de Janeiro, Brazil for molecular tests. All indistinguishable *E. histolytica/E. dispar/E. moshkovskii* complex and *E. hartmanni* positive faecal samples were subjected to DNA extraction using the ZR Fungal/Bacterial DNA MiniPrep™ kit. Nested-polymerase chain reaction (PCR) was performed according to the protocol described by Paglia and Visca (2004). Initially, 1,076 bp fragment of the small subunit rRNA gene sequence common to the *Entamoeba* genus was amplified using primers E1 (5-TGCTGTGATTAAAACGCT-3) and E2 (5-TTAACTATTTCAATCTCGG-3). Nested-PCR was performed with primers Eh-L (5-ACATTTTGAAGACTTTATGTAAGTA-3) and Eh-R (5-CAGATCTAGAAACAATGCTTCTCT-3), which are specific for *E. histolytica* and amplify a 427 bp fragment, Ed-L (5-GTTAGTTATCTAATTTCGATTAGAA-3) and Ed-R (5-ACACCACTTACTATCCCTACC-3), which are specific for *E. dispar* and amplify a 195 bp product, and Mos 1 (5-GAAACCAAGAGTTTCACAAC-3) and Mos 2 (5-CAATATAAGGCTTGGATGAT-3), which are specific for *E. moshkovskii* and yield a 553 bp product (Paglia & Visca 2004, Lau et al. 2013). Molecular characterisation of *E. hartmanni* was performed essentially as described by Gomes et al. (2014), but with minor modifications. Briefly, primers EhartR1 mod (5-ATTGTCTTCACTATTCCATGCC-3) and EhartF mod (5-CCAGCTTTCCAAACATGATG-3) were used to amplify a 186 bp product. PCR products were resolved on 1.5% agarose gels, stained with ethidium bromide, and visualised*via* ultraviolet illumination.

*Ethics* - This study was approved by the Ethical Committee in Research with Humans, Oswaldo Cruz Institute, Oswaldo Cruz Foundation (CAAE: 12125713.5.0000.5248).

## RESULTS

**Fig. 2:**
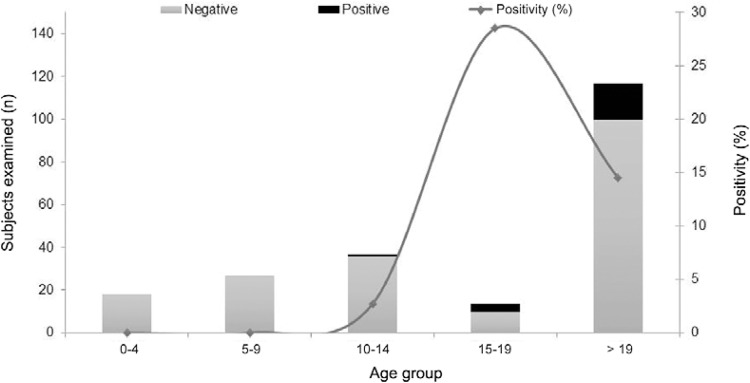
: results of parasitological analysis for *Entamoeba histolytica*/*Entamoeba dispar*/*Entamoeba moshkovskii*complex *and Entamoeba hartmanni*considering the age groups (Russas, state of Ceará, Brazil, 2013).

The overall prevalence of infection with indistinguishable *E. histolytica/E. dispar/E. moshkovskii* complex and *E. hartmanni*organisms was 22/213 (10.3%). Of these 22 positive faecal samples, one was identified only through the flotation (Faust) method, 13 were identified only with the centrifugation (Ritchie 1948) method, and eight were identified with both techniques. The detection rates of nonpathogenic amoebas were as follows: *Endolimax nana*, 4.2% (n = 9),*Entamoeba coli*, 11.3% (n = 24), and *Iodamoeba butschlli*, 7% (n = 15). *Giardia intestinalis* was detected in 30 subjects (14.1%). The age distribution of indistinguishable*E. histolytica/E. dispar/E. moshkovskii* complex and *E. hartmanni* infections is presented in Fig. 2. Regarding infection positivity according to sex, indistinguishable *E. histolytica/E. dispar/E. moshkovskii* complex and *E. hartmanni* infections were found in 12/106 males and 10/107 females (p = 0.704).

As presented in Table II, the detection rate of indistinguishable *E. histolytica/E. dispar/E. moshkovskii*complex and *E. hartmanni* among subjects who drink rainwater collected from roofs in tanks was higher than the rate in people who drink desalinated water pumped from wells. In addition, the detection rate among subjects who practice open defecation was significantly higher than that of inhabitants who have latrines. The positivity rates of subjects in different income strata were similar.

**TABLE II:** Rate of detection of *Entamoeba histolytica*/*Entamoeba dispar*/*Entamoeba moshkovskii* complex and*Entamoeba hartmanni* by source of drinking water, place of defecation, and income, Russas, state of Ceará, Brazil, 2013

	Positive/tested subjects (% of positive)	p^*a*^
Source of drinking water		
Desalinated brackish water from wells	9/138 (6.5)	0.054
Rain water stored in cisterns	9/56 (16.1)	
Sanitation facilities		
Latrine	13/166 (7.8)	0.032
Open defecation	9/47 (19.1)	
Family month income per capita		
< US$^*b*^ 17	4/20 (20)	0.308
US$ 17-34	2/27 (7.4)	
> US$ 34	16/166 (9.6)	

*a*: Fisher exact test; *b*: US$ 1.00 = R$ 4,00 (22 September 2015).

**Fig. 3:**
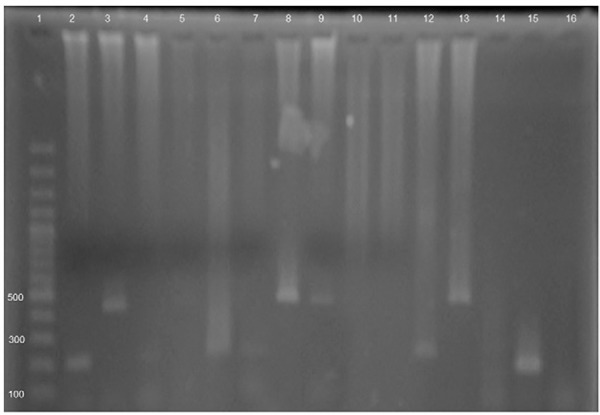
: detection and differentiation of *Entamoeba histolytica*, *Entamoeba moshkovskii*,*Entamoeba dispar* and *Entamoeba hartmanni* by nested-polymerase chain reaction. PCR products were visualised in 1.5% agarose gel with EtBr staining. Line 1: 100 bp DNA ladder; 2, 3: one faecal sample with mixed infection by *E. dispar* and *E. histolytica*, respectively; 4, 6, 7: faecal samples positive for *E. dispar*; 5, 14: empty wells; 8, 9: faecal samples positive for *E. histolytica*; 10: negative control for *E. dispar*; 11: negative control for *E. histolytica*; 12: positive control for *E. dispar*; 13: positive control for *E. histolytica*; 15: faecal sample positive for *E. hartmanni*; 16: negative control.

**Fig. 4:**
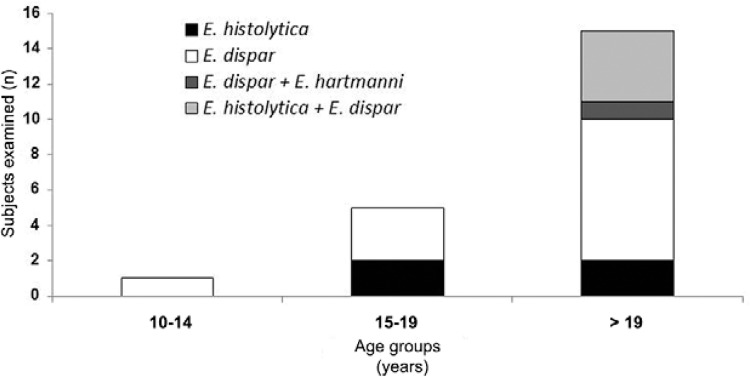
: frequency of identification of *Entamoeba histolytica*, *Entamoeba moshkovskii*,*Entamoeba dispar* and *Entamoeba hartmanni* by nested-polymerase chain reaction by age group in 21 positive subjects (Russas, state of Ceará, Brazil, 2013).

Species-level identification could be performed for 21 of the 22 samples positive for indistinguishable *E. histolytica/E. dispar/E. moshkovskii* complex and *E. hartmanni.* The species distribution was as follows: 12 (57.1%) *E. dispar*, 5 (23.8%) *E. histolytica*, 3 (14.3%) co-infections with *E. histolytica* and *E. dispar*, and one (4.8%) co-infection with *E. dispar* and*E. hartmanni* (Fig. 3). No sample was positive for *E. moshkovskii*. The age distributions of subjects infected with different species are shown in Fig. 4.

## DISCUSSION

A key issue for understanding the morbidity associated with amoebiasis is to define the proportion of infections associated with the pathogenic species *E. histolytica*. Interestingly, studies in different regions have shown that many subjects infected with indistinguishable *E. histolytica/E. dispar/E. moshkovskii* complex and *E. hartmanni*parasites actually harbour low-pathogenicity species such as *E. dispar*, *E. moshkovskii*, or even *E. hartmanni* (Gomes et al. 2014,Nair & Variyam 2014, Efunshile et al. 2015, , Nath et al. 2015). The proportions of these subjects are variable, but can be quite high.

*E. dispar* and *E. moshkovskii* are indistinguishable from *E. histolytica* by light microscopy. Thus, routine parasitological techniques are not suitable for discriminating these organisms. This limitation means that a significant number of patients being treated with antiparasitic drugs such as metronidazole may not actually be infected with*E. histolytica*.

In the present study, approximately two-thirds of all infections were not caused by*E. histolytica*. We note that all subjects were asymptomatic at the time of the stool test. Even so, we infer that nonpathogenic species are detected more frequently than *E. histolytica* in the studied area. This observation is particularly relevant because increasing importance has been given to traditionally nonpathogenic species such as *E. dispar* and*E. moshkovskii*, since invasive amoebiasis has been demonstrated to be associated with these species (Parija & Khairnar 2005). It is likely that the determinants of invasive amoebiasis are complex and also involve host factors (Bosch & Siderovski 2013, Thibeaux et al. 2013).

The nonpathogenic species *E. hartmanni* can be distinguished from*E. histolytica*, *E. dispar*, and *E. moshkovskii* by light microscopy. However, this distinction requires detailed observation of nuclear structures, which requires permanent smear staining, an ocular micrometer, and a highly skilled parasitologist. These criteria are hard to meet for many laboratories. We propose that the possibility of *E. hartmanni* infection should also be considered in people who excrete indistinguishable *E. histolytica/E. dispar/E. moshkovskii* complex and *E. hartmanni* cysts. In the present study, *E. hartmanni* was detected in one of the indistinguishable *E. histolytica/E. dispar/E. moshkovskii* complex and *E. hartmanni* positive samples.

The study population is located in a sociodemographic and environmental setting characterised by deficits in sanitation infrastructure and water stress. The study area is located in a low-rainfall region in the *Caatinga* biome that is subjected to prolonged droughts and prone to desertification. Nonpotable water is obtained from a reservoir in the locality and used for livestock watering and other suitable applications. In the last decade, a strategy has been implemented in which rainwater is collected during the rainy season from roofs *via*gutters. This collected rainwater is stored in household tanks for later use during droughts. This strategy has significantly improved access to drinking water in the study area. Artesian wells constructed in the region are another source of drinking water. However, this water is brackish and must be desalinated before consumption. We found that the rate of *E. histolytica*, *E. dispar*, and *E. hartmanni* positivity was almost three times higher in subjects who drink collected rainwater than in subjects who drink desalinated brackish water drawn from the artesian wells. We hypothesise that the long period (between the dry season and the rainy season) of rainwater storage in tanks favours contamination with amoeba cysts, thereby enabling transmission. Interestingly, consumption of rainwater captured from roofs has been demonstrated to reduce the prevalence of *G. intestinalis* infection in a semiarid region in northeastern Brazil (Fonseca et al. 2014). Regarding the place of defecation, subjects who practice open defecation exhibited a significantly higher positive rate compared with subjects who defecate in latrines. Moreover, an even higher positive rate was observed in people who deposit faeces directly into the soil compared with subjects with rudimentary tanks.

In some regions of the world, including Latin America, inadequate sanitary conditions facilitate the transmission of amoebiasis, thereby generating high prevalence rates (Braga et al. 1998, Ramos et al. 2005). In these scenarios, invasive amebic dysentery and liver abscesses are expected to occur. However, these diseases were not observed in the present study. Severe cases of amoebiasis are identified infrequently in Brazil, which may be explained by the relative improvement of living conditions over the past few decades.

Cumulatively, our data suggest a high prevalence of asymptomatic infection with indistinguishable *E. histolytica/E. dispar/E. moshkovskii* complex and *E. hartmanni* parasites. These asymptomatic infections appear to be caused by predominantly nonpathogenic species or parasites with low pathogenic potential. In the context of scarce water resources, the sanitary and socioenvironmental characteristics of the region appear to be associated with transmission.
